# Correlation of pain relief with physical function in hand osteoarthritis: randomized controlled trial *post hoc *analysis

**DOI:** 10.1186/ar2906

**Published:** 2010-01-11

**Authors:** H Richard Barthel, John H Peniston, Michael B Clark, Morris S Gold, Roy D Altman

**Affiliations:** 1 Private Practice (Rheumatology), P.O. Box 30813, Santa Barbara, CA 93130, USA; 2 Feasterville Family Health Care Center, 1665 Bustleton Pike, Feasterville, PA 19053, USA; 3 Endo Pharmaceuticals Inc., 100 Endo Boulevard, Chadds Ford, PA 19317, USA; 4 Novartis Consumer Health, Inc., 200 Kimball Drive, Parsippany, NJ 07054, USA; 5 Department of Rheumatology and Immunology, David Geffen School of Medicine, University of California, 1000 Veteran Avenue, Los Angeles, CA 90024, USA

## Abstract

**Introduction:**

Nonsteroidal anti-inflammatory drugs are recommended for the relief of pain associated with hand osteoarthritis (OA) but do not alter the underlying structural changes that contribute to impaired physical function. The current analysis examined the relationship of pain relief with measures of function and global rating of disease in patients with hand OA.

**Methods:**

This was a combined analysis of 2 prospective, randomized, double-blind, 8-week, multicenter, parallel-group studies comparing diclofenac sodium 1% gel with placebo gel (vehicle) in patients with radiographically confirmed mild to moderate hand OA. Patients (n = 783) aged ≥ 40 years applied diclofenac sodium 1% gel (2 g) or vehicle to each hand 4 times daily for 8 weeks. Outcome measures included pain intensity assessed on a 100-mm Visual Analog Scale (VAS); the Australian/Canadian Osteoarthritis Hand Index (AUSCAN) subscales for pain, stiffness, and physical function (100-mm VAS); and a global rating of disease (100-mm VAS). Change in VAS pain intensity from baseline to week 8 was categorized (<0%, 0%-<15%, 15%-<30%, 30%-<50%, 50%-<70%, and ≥ 70%) without regard to treatment and compared in each category with the mean change from baseline in each AUSCAN subindex and the global rating of disease. Pearson correlations between changes in outcome measures from baseline to week 8 were calculated.

**Results:**

Changes in VAS pain intensity were accompanied by similar changes in AUSCAN scores and global rating of disease. Pearson correlations confirmed significant associations (*P *< 0.001) between change in VAS pain intensity and changes in AUSCAN pain (correlation coefficient [*r*] = 0.81), AUSCAN function (*r *= 0.75), AUSCAN stiffness (*r *= 0.66), and global rating of disease (*r *= 0.76).

**Conclusions:**

Pain relief correlated with improvements in physical function, stiffness, and global rating of disease in patients with hand OA, irrespective of treatment. This suggests that pain or anticipation of pain inhibits physical function and influences patient perception of disease severity in hand OA. These results also suggest that any intervention to relieve the pain of hand OA may improve function and patient perception of disease severity, despite the absence of a disease-modifying mechanism of action.

**Trial registration:**

Clinicaltrials.gov NCT00171652, NCT00171665.

## Introduction

Hand osteoarthritis (OA) has an estimated prevalence of 20% to 30% [1,2], making the hand the second most frequent site of OA pain [1,3]. The prevalence of hand OA increases with age, surpassing 50% after patients reach the age of 60 years [4-6]. Symptoms include not only pain but also functional impairment in the form of stiffness, reduced grip strength, reduced hand mobility, and difficulty performing dexterous tasks [2,4,7,8].

Function is irreversibly compromised in OA of the hand as articular surfaces are eroded and deformed. In OA of the knee and hip, a definitive improvement in function can be obtained with surgical replacement of the joint, but prosthetic joints have been less successful for hand OA [9]. More often, surgery for hand OA may be performed for cosmetic reasons rather than to provide functional improvement (for example, patients self-conscious of and eager to remove Heberden nodes).

Nonsteroidal anti-inflammatory drugs (NSAIDs) are recommended for the management of pain in patients with hand OA who do not respond to physical measures and acetaminophen [10]. Though effective for the treatment of mild to moderate OA pain [11], NSAIDs have been associated with an increase in the risk of serious gastrointestinal adverse events, including ulcers, perforations, and bleeding related to dose and duration of use [12,13]. The potential risk of cardiovascular [14-16] and renal [17,18] adverse events with NSAIDs is also considered exposure-related and generally observed during long-term NSAID therapy.

Treatment guidelines recommend topical NSAIDs as effective monotherapy for relief of OA pain in superficial joints, such as those in the hands [10], with the potential to mitigate the risk of NSAID-related adverse events by reducing systemic NSAID exposure. Topical diclofenac sodium 1% gel provided safe and effective pain relief compared with placebo in a large clinical trial in patients with hand OA [19]. Administration of diclofenac sodium 1% gel results in substantially lower systemic diclofenac concentrations than occur following oral administration [20].

NSAIDs relieve OA pain but are not believed to alter the underlying changes that produce biomechanical limitations of physical function in OA. However, other interventions that provide symptomatic relief without altering underlying structural changes, such as opioids, have been associated with improvement of physical function in OA of the knee and hip [21]. This finding suggests that in addition to the biomechanical limitations caused by hypertrophic changes in OA, it is possible that pain or the anticipation of pain leads to voluntary and involuntary restriction of activity [22]. If this is true, relief of pain alone may improve physical function in OA although no biomechanical improvement has occurred. In the present analysis, we tested the hypothesis that pain relief is associated with improved physical function in patients with hand OA.

## Materials and methods

### Study design

This was an analysis of pooled data from two similar 8-week, randomized, double-blind, parallel-group, multicenter trials comparing diclofenac sodium 1% gel with vehicle gel in patients with mild to moderate hand OA. Efficacy and safety results for one of these studies have been presented elsewhere [19]. Ethical approval was obtained from an independent ethics committee or institutional review board for all participating study sites (Ärztekammer Berlin for study sites in Germany, CCPPRB de Paris Pitié Salpétrière for study sites in France, and Quorum Review, Inc. [Seattle, WA, USA] for study sites in the US). The studies were conducted in accordance with the Declaration of Helsinki, Directive 91/507/EEC of the Rules Governing Medicinal Products in the European Community, and US 21 Code of Federal Regulations (parts 50 and 56) dealing with clinical studies. All patients provided written informed consent before participating.

### Patients

Patients included men and women at least 40 years old who had a diagnosis of primary hand OA by American College of Rheumatology criteria [23], with symptoms including pain for at least 12 months previously, radiographically confirmed to be of mild to moderate severity (Kellgren-Lawrence grades 1 to 3). At screening, patients had to have pain more than 15 days during the previous 30 days and must have had at least one painful episode treated with an NSAID or salicylate during the previous year. Patients also had to be able to indicate right- or left-handed dominance and to have reported that pain was greater in the dominant hand, which was the target hand. At baseline, patients had to have pain in their target hand during the previous 24 hours rated at least 40 mm on a 100-mm Visual Analog Scale (VAS). If washed out from NSAIDs, patients had to have an at least 15-mm pain increase between the screening and baseline visits. Pain scores had to be at least 20 mm lower in the nondominant hand relative to the dominant hand.

Main exclusion criteria were secondary post-traumatic OA, history or evidence (or both) of any other rheumatic disease involving the potential target hand or the arm, symptomatic OA at additional locations besides the hand(s) requiring any symptomatic or disease-modifying drug, adult juvenile chronic arthritis (that is, juvenile chronic arthritis with continued activity in adulthood), history of rheumatoid arthritis or laboratory values indicative of rheumatoid arthritis, history of other inflammatory diseases (such as colitis) within the previous year, or a history of fibromyalgia within the previous year.

### Randomization and treatment regimen

After screening, patients meeting inclusion criteria washed out previous analgesics for 1 week or at least five half-lives of the previous analgesic, whichever was longer. After washout, patients were randomly assigned (1:1 ratio) in a double-blind fashion to treatment with diclofenac sodium 1% gel (Voltaren^® ^Gel; Endo Pharmaceuticals Inc., Chadds Ford, PA, USA) or vehicle. To that end, randomization numbers were allocated to centers in blocks to balance treatment allocation within each center.

Patients were assigned to receive 4 g diclofenac sodium 1% gel (2 g to each hand) or vehicle gel four times daily for 8 weeks. Diclofenac sodium 1% gel contains diclofenac sodium and its vehicle, which consists of isopropyl alcohol, propylene glycol, cocoyl caprylocaprate, mineral oil, ammonia solution, perfume cream 45/3, carbomer homopolymer type C, polyoxyl 20 cetostearyl ether, and purified water. Vehicle gel was identical to diclofenac sodium 1% gel in composition but did not include the active ingredient, diclofenac sodium. The two gels were identical in feel, appearance, and smell. Treatments were dispensed in kits containing six 50-g tubes of study medication (diclofenac sodium gel or vehicle) for a 2-week supply. Use of acetaminophen rescue medication was allowed up to a maximum daily dose of 4 g. However, rescue medication was not allowed within 36 hours of assessments. Disease-modifying drugs, muscle relaxants, additional analgesics, and alternative therapies (for example, acupuncture) were not allowed.

### Assessments

Measures of pain intensity were compared with measures of physical function and patient global rating of disease in all patients. Pain intensity in the dominant hand was assessed on a 100-mm VAS (VAS pain intensity, 0 = no pain, 100 = unbearable pain). Pain, stiffness, and function were also assessed using the Australian/Canadian Osteoarthritis Hand Index (AUSCAN), a 15-item tool that focuses on the functional status of the dominant hand. Each AUSCAN item was assessed on a 100-mm VAS. Total AUSCAN scores were calculated for pain (questions 1 to 5), stiffness (question 6), and function (questions 7 to 15) as averages over the individual questions. A global rating of disease was assessed on a 100-mm VAS (0 = very good; 100 = very poor). For this assessment, patients responded to the following item: 'Considering all the ways osteoarthritis of your hands affects you, please indicate with an 'X' through the horizontal line how well are you doing'.

### Statistical analysis

The purpose of the present analysis was to determine whether changes in pain were accompanied by similar changes in function rather than to compare diclofenac sodium 1% gel with placebo. To that end, data on all patients who received at least one dose of study medication were pooled without regard to whether they were assigned diclofenac sodium 1% gel or placebo. Improvement in VAS pain intensity from baseline to week 8 was categorized according to percentage change (less than 0%, 0% to less than 15%, 15% to less than 30%, 30% to less than 50%, 50% to less than 70%, and 70% or more) without regard to randomized treatment. Within each VAS pain intensity improvement category, AUSCAN pain, AUSCAN stiffness, and AUSCAN function subindices and global rating of disease were summarized by mean change from baseline to week 8. Mean percentage change from baseline to week 8 was computed by category as the mean change from baseline divided by the mean baseline value. The associations between change from baseline for VAS pain intensity and changes from baseline for the three AUSCAN subindices and the global rating of disease were also summarized as Pearson correlations. Postbaseline assessments were conducted at weeks 1, 2, 4, 6, and 8. For patients who terminated prematurely, the final available assessment was used instead of the week 8 assessment.

## Results

### Patients

**Figure 1:**
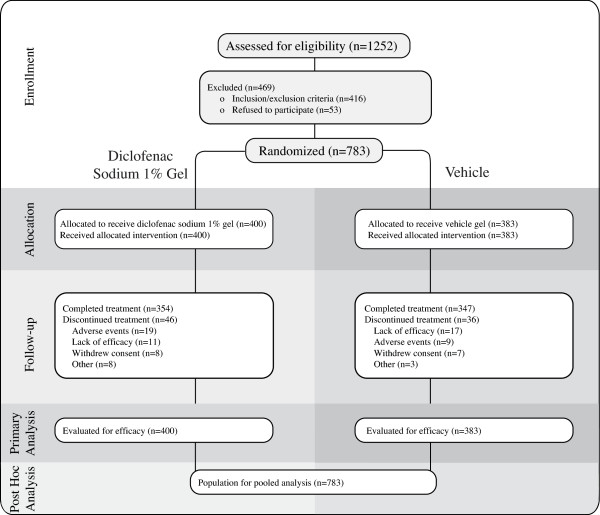
**CONSORT diagram describing study pooling and post-hoc analysis in relation to original study design**.

Of 1,252 patients screened, 783 were randomly assigned to receive diclofenac sodium 1% gel (n = 400) or vehicle (n = 383) (Figure 1). In all, 701 (89.5%) patients completed the study. All 783 were included in the correlation analysis. The majority of patients were female (80.2%) and white (93.6%) (Table 1). Mean (standard deviation [SD]) VAS pain intensity (71.1 [15.1] mm) was moderate to severe. Mean scores on the three AUSCAN subindices and global rating of disease also reflected moderate pain, functional impairment, and impact of disease on patients' sense of well-being. Nearly half of the patients (46.1%) had Kellgren-Lawrence grade 3 OA, indicating moderate rather than mild anatomic disease (Table 2), but baseline mean VAS pain intensity scores were similar (approximately 70 mm) for each grade. In addition, mean changes from baseline through week 8 in VAS pain intensity, on the three AUSCAN subindices, and global rating of disease were similar for patients with Kellgren-Lawrence grades 1, 2, and 3. Most patients had radiographic evidence of sclerosis (63.3%), joint space narrowing (78.0%), and osteophytes (78.3%). More than a third (38.6%) had subchondral cysts. A majority (58%) had a pain reduction of at least 30%. At least 10% of patients fell within each of the pain reduction categories (Table 3).

**Table 1:** Baseline demographics and assessments

	Diclofenac sodium 1% gel	Vehicle	Combined
	n = 400	n = 383	n = 783
Percentage of female patients	79.3	81.2	80.2
Percentage of white patients	93.0	94.3	93.6
Age, years			
Mean (SD)	63.8 (10.0)	64.1 (9.7)	63.9 (9.8)
Range	40-92	40-87	40-92
Body mass index, kg/m^2^			
Mean (SD)	27.5 (5.4)	27.5 (5.6)	27.5 (5.5)
Range	17.4-55.0	14.3-49.8	14.3-55.0
VAS pain intensity			
Mean (SD)	71.1 (15.3)	71.1 (14.8)	71.1 (15.1)
Range	26-100	4-100	4-100
AUSCAN pain			
Mean (SD)	63.9 (17.5)	63.4 (17.7)	63.7 (17.6)
Range	11.6-98.4	10.4-99.0	10.4-99.0
AUSCAN stiffness			
Mean (SD)	60.4 (24.7)	59.7 (25.7)	60.0 (25.2)
Range	1-98	0-100	0-100
AUSCAN function			
Mean (SD)	65.9 (18.1)	64.6 (18.8)	65.3 (18.4)
Range	8.9-98.7	8.3-99.2	8.3-99.2
Global rating of disease			
Mean (SD)	56.9 (18.1)	56.7 (18.7)	56.8 (18.4)
Range	5-97	9-98	5-98

AUSCAN, Australian/Canadian Osteoarthritis Hand Index; SD, standard deviation; VAS, Visual Analog Scale.

**Table 2:** Baseline radiographic evaluations

Disease characteristic	Diclofenac sodium 1% gel	Vehicle	Combined
	n = 400	n = 383	n = 783
Kellgren-Lawrence grade			
1	70 (17.5)	54 (14.1)	124 (15.8)
2	141 (35.2)	157 (41.0)	298 (38.1)
3	189 (47.3)	172 (44.9)	361 (46.1)
Sclerosis	251 (62.7)	245 (64.0)	496 (63.3)
Subchondral cysts	153 (38.2)	149 (38.9)	302 (38.6)
Joint space narrowing	317 (79.2)	294 (76.8)	611 (78.0)
Osteophytes	305 (76.2)	308 (80.4)	613 (78.3)

Values are presented as number (percentage).

**Table 3:** Frequency distribution for pain intensity improvement

Pain reduction category	Patients, number (percentage)
Less than 0%	85 (10.9)
0% to less than 15%	158 (20.2)
15% to less than 30%	83 (10.6)
30% to less than 50%	100 (12.8)
50% to less than 70%	110 (14.0)
70% or more	247 (31.5)

Total	783 (100.0)

### Efficacy

**Figure 2:**
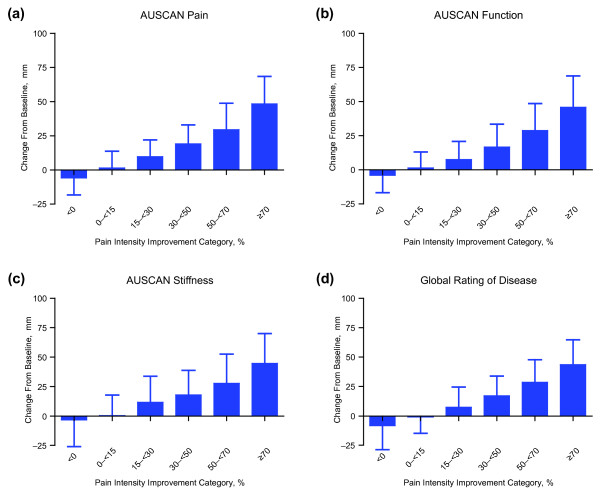
**Mean (standard deviation) changes in (a) Australian/Canadian Osteoarthritis Hand Index (AUSCAN) pain, (b) AUSCAN function, (c) AUSCAN stiffness, and (d) global rating of disease from baseline to week 8 by pain intensity category**.

Mean changes from baseline in AUSCAN and global rating of disease scores were consistent with changes in VAS pain intensity (Figure 2). When VAS pain intensity scores improved, AUSCAN subscale scores and global rating of disease showed similar improvement. At one extreme, patients showing little or no improvement (0% to less than 15%) in VAS pain intensity had little or no mean (SD) improvement in AUSCAN pain (1.5 [12.2] mm; 2.2%), AUSCAN function (1.4 [11.7] mm; 1.9%), AUSCAN stiffness (0.4 [17.5] mm; 0.6%), and global rating of disease (-0.8 [13.8] mm; -1.3%). At the other extreme, patients with at least 70% improvement in VAS pain intensity had large mean (SD) improvements in AUSCAN pain (48.3 [20.1] mm; 76.9%), AUSCAN function (45.9 [22.9] mm; 71.2%), AUSCAN stiffness (44.9 [25.5] mm; 75.2%), and global rating of disease (43.8 [21.1] mm; 78.1%). Patients whose VAS pain intensity worsened over the 8-week treatment period also experienced worsening of mean (SD) scores for AUSCAN pain (-5.8 [12.5] mm; -8.8%), AUSCAN function (-4.0 [12.6] mm; -5.9%), AUSCAN stiffness (-3.3 [22.7] mm; -5.4%), and global rating of disease (-8.22 [20.4] mm; -14.2%).

Pearson correlations confirmed the association between change in VAS pain intensity and changes in AUSCAN scores and global rating of disease (Table 4). Change in VAS pain intensity was found to be highly correlated (*P *< 0.001) with AUSCAN pain (correlation coefficient [*r*] = 0.81), AUSCAN function (*r *= 0.75), AUSCAN stiffness (*r *= 0.66), and global rating of disease (*r *= 0.76).

**Table 4:** Correlations between changes in outcome measures from baseline to week 8

	VAS pain intensity	AUSCAN pain	AUSCAN stiffness	AUSCAN function	Global rating of disease
VAS pain intensity	1.00	0.81^a^	0.66^a^	0.75^a^	0.76^a^
AUSCAN pain		1.00	0.74^a^	0.88^a^	0.75^a^
AUSCAN stiffness			1.00	0.75^a^	0.60^a^
AUSCAN function				1.00	0.71^a^
Global rating of disease					1.00

^a^*P *< 0.001. AUSCAN, Australian/Canadian Osteoarthritis Hand Index; VAS, Visual Analog Scale.

## Discussion

In this analysis, we found that improvements in pain in patients treated with diclofenac sodium 1% gel or vehicle for symptomatic hand OA were accompanied by similar improvements in measures of functional status and patients' overall rating of OA disease status. Our study population included patients with mild to moderate hand OA (Kellgren-Lawrence grades 1 to 3), most of whom had structural changes that typically contribute to functional impairment in OA. Mean VAS pain intensity was moderate to severe.

The similarity of mean changes in pain and functional impairment scores that we observed across Kellgren-Lawrence severity grades 1, 2, and 3 is consistent with research showing that radiographic findings in hand OA are only modestly associated with reports of pain and weakly associated with functional disability [4]. The strong association observed between pain and function in our study suggests an important role for symptomatic treatment in improving the functional status of patients with hand OA of mild to moderate radiographic severity.

Impairment of joint function in patients with hand OA is of great concern because of its impact on many activities of daily living and almost every type of employment. Unfortunately, effective disease-modifying interventions to slow or reverse the structural changes that impair function in hand OA have not yet been found. Modest results have been obtained in some studies of chondroitin sulfate [24-26] and doxycycline [27]. Although surgical interventions of joint replacement and resurfacing of the knee or hip have been quite successful [28] and associated with improved function and quality of life [29], outcomes in smaller joints, such as those in the hand, have been less successful [30-33]. In the absence of modalities to prevent or reverse the structural changes contributing to functional impairment in hand OA, it is important to determine whether relief of pain alone can improve function in this population.

The observed correlation in our analysis between reductions in pain and improvements in function suggests that some functional restriction associated with hand OA stems from patients voluntarily or involuntarily restricting activity because of perceived or anticipated pain and not solely from the biomechanical limitations of affected joints. Neither diclofenac sodium 1% gel nor its vehicle is believed to influence structural changes in hand OA or provide any disease-modifying benefit. This implies that the observed improvements in functional outcomes and health status occurred in the absence of structural improvement.

The results of this analysis are consistent with a previous study that showed an association between pain and both functional and overall health status in patients with hand OA [34] and OA of the knee and hip [21]. In a study assessing the validity of the AUSCAN hand index, all three AUSCAN subindices were significantly correlated with an independent measure of pain and with grip strength [34]. As in the present study, the independent pain measure was most strongly correlated with the AUSCAN pain subindex, but correlations with the function and stiffness subindices were nearly as strong.

Our results are also consistent with two trials assessing patients with OA of the knee or hip. In a 12-week placebo-controlled trial of the long-acting opioid tramadol extended release [21], reductions in pain associated with knee or hip OA were significantly correlated with improvements in measures of function (the Western Ontario and McMaster Universities Osteoarthritis Index) and health status (the Short Form-36 Health Survey [SF-36]). Similarly, in a study of diclofenac sodium 1% gel, pain reduction in knee OA was accompanied by and significantly correlated with improvements in Western Ontario and McMaster Universities Osteoarthritis Index stiffness and physical function indices and in a global rating of disease [35]. As in our study, patients in these studies received no concurrent disease-modifying drugs, reinforcing the idea that observed effects of pain reduction on functional outcomes and health status occurred in the absence of structural improvement.

Our analysis differed from the study of tramadol in that we assessed pain and function in hand OA and evaluated health status using a global rating of disease instead of the SF-36. SF-36 is a broad rating of overall health status, whereas the global rating of disease specifically asks patients to rate the impact of OA on their daily lives. Thus, our results establish a link between pain, physical function, and patients' sense of how well they are coping with hand OA.

Current guidelines for the management of hand OA do not recommend opioids but do suggest that studies be conducted to assess and compare the efficacy of acetaminophen, weak opioids (such as tramadol), and oral NSAIDs in this population [10]. According to these guidelines, local treatments, such as topical NSAIDs, should be considered for the management of pain, particularly in patients who have mild to moderate OA. Results from the present analysis suggest that pain reductions during treatment with a topical therapy may also be accompanied by improvements in functional status and general sense of well-being in patients with mild to moderate hand OA.

A limitation of our study is that it was only 8 weeks. Hand OA is a chronic disease that may require treatment over several decades. Long-term trials would be necessary to determine whether the association between analgesia and functional improvement is maintained over time. Another limitation of our study is that we have not considered possible variations in response between subpopulations with different affected hand joints (for example, first carpometacarpal versus interphalangeal OA). Finally, because this was a *post hoc *analysis, the results should be considered exploratory. The validity of the results would be strengthened if similar correlations could be found in other studies employing a variety of designs, especially those with alternative measures of pain and function, conducted for other purposes.

## Conclusions

Improvements in the pain of hand OA were associated with substantial improvements in physical function, stiffness, and overall rating of OA disease status, without regard to active versus placebo treatment. Diclofenac sodium 1% gel is indicated for relief of OA pain in joints amenable to topical treatment, such as the hands and knees. The placebo gel has no therapeutic indication. This suggests that pain or anticipation of pain inhibits physical function and influences patient perception of disease severity in hand OA. These results also suggest that any intervention to relieve the pain of hand OA may improve function and patient perception of disease severity, despite the absence of a disease-modifying mechanism of action.

## Abbreviations

AUSCAN: Australian/Canadian Osteoarthritis Hand Index; NSAID: nonsteroidal anti-inflammatory drug; OA: osteoarthritis; *r*: correlation coefficient; SD: standard deviation; SF-36: Short Form-36 Health Survey; VAS: Visual Analog Scale.

## Competing interests

HRB and JHP declare that they have no competing interests. MBC is a full-time employee of Endo Pharmaceuticals Inc. MSG is a full-time employee of Novartis Consumer Health, Inc. (Parsippany, NJ, USA). RDA has received research grants from Novartis Consumer Health, Inc. and Ferring Pharmaceuticals, Inc. (Parsippany, NJ, USA) and consulting fees from Novartis Consumer Health, Inc., Ferring Pharmaceuticals, Inc., and Rottapharm (Monza, Italy) and has participated in speakers' bureaus for Ferring Pharmaceuticals, Inc. and Forest Laboratories, Inc. (New York, NY, USA).

## Authors' contributions

RDA contributed to the conception and design of the original randomized clinical trials and helped to provide some of the study subjects for the original randomized clinical trials used in this *post hoc *analysis. MSG contributed to the conception and design of this *post hoc *analysis and provided statistical expertise. MBC contributed to the conception and design of this *post hoc *analysis and obtained funding. HRB and JHP helped to provide some of the study subjects for the original randomized clinical trials used in this *post hoc *analysis. All authors contributed to the analysis and interpretation of the data, drafting of the article, critical revision of the article for important intellectual content, and final approval of the article. All authors read and approved the final manuscript.
